# Ecotoxicity Assessment of Graphene Oxides Using Test Organisms from Three Hierarchical Trophic Levels to Evaluate Their Potential Environmental Risk

**DOI:** 10.3390/nano13212858

**Published:** 2023-10-28

**Authors:** Imre Németh, Krisztina László, Anna Bulátkó, Emese Vaszita, Mónika Molnár

**Affiliations:** 1 Department of Applied Biotechnology and Food Science, Faculty of Chemical Technology and Biotechnology, Budapest University of Technology and Economics, H-1111 Budapest, Hungary; nemeth.imre@vbk.bme.hu (I.N.); vaszita.emese@vbk.bme.hu (E.V.); 2 Department of Physical Chemistry and Materials Science, Faculty of Chemical Technology and Biotechnology, Budapest University of Technology and Economics, H-1111 Budapest, Hungary; laszlo.krisztina@vbk.bme.hu (K.L.); bulatko.anna@edu.bme.hu (A.B.)

**Keywords:** ecotoxicity, environmental risk assessment, graphene oxide, nanomaterials, predicted no-effect concentration

## Abstract

After more than a decade of studying the ecotoxicity of graphene oxide nanomaterials (nGOs), it has been concluded that there is limited information available regarding the environmental risk of graphene-based materials. Since existing ecotoxicological studies of nanomaterials have produced contradictory results, it is recommended that case-by-case studies should be conducted to evaluate their effects. This can be carried out by employing several methods, testing species from different trophic levels, and conducting community studies. Our goal was to evaluate the toxicity effects of two GOs (AF 96/97 and PM 995) derived from different graphite precursors on various test organisms from diverse trophic levels (bacteria, protozoa, a freshwater microbial community, plants, and invertebrate animals) in aquatic environments. We compared the effects of both nGO types and estimated the predicted no-effect environmental concentration (PNEC) values to determine their potential environmental risk. Our findings demonstrated the need for a complex ecotoxicity toolkit since the ecotoxicity results varied based on the test organism, the selected endpoints, and the test method used. Additionally, we found that toxicity effects were dependent on the concentration and characteristics of the specific nGO type used, as well as the exposure time. We estimated the PNEC values for GO AF 96/97 and GO PM 995 in the aquatic compartment to be 8 ng/L and 4 ng/L, respectively. Even after applying the worst-case scenario approach, the tested nGOs pose no environmental risk.

## 1. Introduction

Nanoparticles (NPs) are widespread in the environment due to their broad range of applications. They have unique general properties (particle size, shape, charge, surface area, and surface reactivity) relative to their bulk or dissolved counterparts. These characteristics enable a wide range of uses, including medical, pharmaceutical, cosmetic, electrical, and agricultural utilisation; therefore, a significant number of products contain or require nanoparticles for their production [1,2].

Carbon-based nanomaterials include fullerenes, carbon nanotubes, graphene and its derivatives such as graphene oxide, nanodiamonds, carbon-based quantum dots, and amorphous carbon NPs. Due to their unique structural dimensions and excellent mechanical, electrical, thermal, optical, and chemical properties, these materials have attracted significant interest in diverse areas [3]. Graphene has garnered attention in the field of electromagnetics due to its dynamic tunability. The valence bands and conduction of this two-dimensional honeycomb carbon material converge at the Dirac point, thus rendering graphene a semiconductor with a distinctive zero-band-gap structure. Graphene-based absorbers can be flexibly regulated in practical applications, which provides new possibilities for the development of many fields, such as detection and communication. Changes in the top structure parameters have an influence on the absorption performance. Tunable absorbers possess the capability to adapt to different electromagnetic environments [4].

Graphene exhibits a fast full-spectrum response to terahertz waves and strong surface plasmon resonance due to its extremely high conductivity and carrier mobility [5]. These characteristics empower graphene-based materials to achieve multiple perfect absorption peaks in the terahertz range with improved spacing between resonant frequencies and modulation bandwidth [6]. At specific resonant frequencies, graphene-based absorbers can achieve perfect absorption [7]. Tang et al. [5] proposed graphene-based metasurfaces that have potential applications in mid-infrared optoelectronic devices, such as optical switches, modulators, and slow-light devices. A multi-frequency broadband absorber structure based on graphene’s surface plasmon resonance was proposed by Chen et al. [8], which has potential value for application in terahertz photoelectric detection, filtering, and electromagnetic shielding.

Graphene oxide (GO), a hydrophilic, oxidised derivative of graphene, is used in biotechnology and medicine for cancer treatment, drug delivery, and cellular imaging. GO’s characteristics make it attractive in other areas such as sensors [9] and even energy storage [10]. GO and its composites hold great promise for their versatile applications in energy storage and conversion and environment protection, including as hydrogen storage materials, photocatalysts for water splitting, materials for removal of air pollutants and water purification, as well as electrode materials for various lithium batteries and supercapacitors [11].

With their widespread application, their presence in the environment, and the risk of potentially adverse effects in natural systems, exposure to GO has also increased across populations [12]. GO nanoparticles (nGOs) detected in air, soil, and water go through a range of physical, chemical, and biological transformations, which will affect their biological effects. Various environmental factors can induce changes in the morphology, microstructure, and surface properties of GO NPs, as well as alter their toxicity through processes such as aggregation, adsorption, REDOX reaction, phototransformation, microbial reduction, and other biotransformation mechanisms [13]. Consequently, the toxicity of graphene oxide NPs depends on their concentration and physicochemical properties (size, zeta potential, surface chemistry, morphology, and sedimentation). Even the type of environmental media (seawater, soil, and airborne fine particulate) may affect the toxicity of NPs [14], but their ecological and health risks are still not fully known and are still being studied.

The effects of graphene oxide nanoparticles on different test organisms from various trophic levels have been reported. The ecotoxicity of nGOs and their derivatives have been investigated in different test systems, including microbes (bacteria, fungi, and algae), planktonic crustaceans, plants, earthworms, fish, and mice [15,16]. The results and outcomes of these studies demonstrate high uncertainties regarding the ecotoxicological effects of GOs; however, the harmful effects of nGOs have been demonstrated. The toxicity of nGOs has been examined in human cells, where enhanced oxidative stress and cyto- and genotoxicity effects were observed [16]. Graphene-based nanomaterials can potentially disrupt the growth of microbial communities and their structure, activity, and diversity. As microbial communities represent the basis of every ecosystem’s health, these adverse effects may have serious environmental consequences [17].

The toxicity of nGOs is size-dependent and is influenced by the properties of the initial graphite. Smaller graphene oxide nanoparticles have more toxic effects than larger ones. The size of nGO sheets depends on the oxidation degree of the initial graphite, which can be modified during the nGO production by modulating the quantity of oxidants and the duration of oxidation, resulting in nGO sheets with various electrical conductivity and size [18].

To assess and characterise the risk of chemicals, the predicted environmental concentrations (PECs) are compared to the predicted no-effect concentrations (PNECs) as proposed by the EU Technical Guidance Document (TGD) [19]. However, this methodology and approach has its own range of applicability and restrictions. It is often used for the first (preliminary) assessment of the environmental risk described in the EU TGD [19]. The predicted environmental concentration (PEC) of various types of nanoparticles differs because their production and utilization levels differ. There is no PEC information for nGOs in all environmental compartments; thus, further modelling investigations are needed [20]. There is some information about the PEC value of carbon-based nanomaterials (e.g., CNTs), which is supposedly similar to nGO concentrations, although the total input production volume of graphene is reported to be 6–7 times higher than of CNTs [21]. The PEC values of CNTs in surface water range between 2 × 10^−5^ and 1.82 ng/L, while in sediments, the values range between 1 × 10^−4^ and 2.66 × 10^−2^ mg/kg [20]. These concentrations are the lowest among the PEC values of TiO_2_, Ag, CuO, and CNT nanoparticles, which is consistent with the lowest production volume among the four NMs [21].

Strong aggregation and sedimentation can be induced by the high surface hydrophobicity of CNTs [22], which is possibly another reason for the low PECs in surface water. The PEC values of NMs in seawater are generally lower than those in freshwater; thus, PECs in seawater sediments are lower than those in freshwater sediments. In sediments, PECs for all the NMs are 2–4 orders of magnitude higher than those in soils [20].

In summary, an analysis of the available ecotoxicity results demonstrated that the ecotoxicity assessments conducted among various nGOs are based mainly on single-species lab tests, while their effects at the community level are understudied. It has also been revealed that despite their widespread detection in different environmental compartments, there is a knowledge gap in the characterisation of the potential environmental risk of nGOs. Moreover, the mechanisms underlying graphene-based nanomaterial-induced toxicity are influenced by the type and characteristics of a given GO nanoparticle.

However, there is a knowledge gap in ecotoxicology and environmental risk assessment with regard to graphene-based nanomaterials’ environmental impacts. It is recommended to evaluate the effects of these materials in more relevant environmental conditions, such as via multispecies exposure in micro- and mesocosm studies [23].

The objective of this study was twofold. First, this study aimed to examine the toxicity of two distinct forms of nGOs on various test organisms from diverse trophic levels in aquatic environments, including microorganisms, microbial communities, protozoa, nematodes, and plants.

Secondly, this study compared the impacts of both types of nGOs and established the effective concentration values for each test organism, aiming to develop the initial PNEC values that aid in the primary risk characterisation of the tested nGOs. We hypothesised that the safety limit determined based on the results of the complex ecotoxicity assessment would indicate that the tested GO derivatives did not pose any risk to the environment.

## 2. Materials and Methods

The toxicity and the mechanism underlying the toxicity of two types of graphene oxide nanoparticles were evaluated using various methods. Different test systems were applied to distinct test organisms from several trophic levels to measure the toxicity of these GO nanoparticles. The effects of these nGOs in single- and multispecies systems were also examined. There were individual tests with bacterial strains, protozoa, nematodes, and plants, while the microbial community of the water of Lake Balaton (Hungary) was tested using multispecies tests.

### 2.1. Properties of the Applied Graphene Oxide Nanoparticles

**Figure 1:**
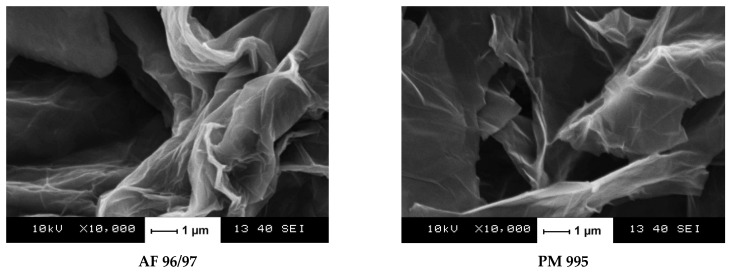
Scanning electron micrographs of the obtained multilayer graphene oxides.

Two commercial graphite precursors, AF 96/97 (carbon content 96–98%, ash content 2–4%, nominal particle size min. 99.5% < 40 μm) and PM 995 (carbon content min. 99.5%, ash content < 0.5%, nominal particle size max. 10% + 0.063 mm), were purchased from Graphit Kropfmühl GmbH (Hauzenberg, Germany) and Graphite Týn (Týn nad Vltavou, Czech Republic), respectively. Wet oxidative exfoliation was performed using a modified Hummers method [24,25]. Briefly, 400 mL of a 9:1 mixture of concentrated H_2_SO_4_/H_3_PO_4_ was added to a mixture containing 3 g of graphite flakes and 18 g of KMnO_4_. The mixture was stirred at 50 °C for 12 h. Then, the mixture was cooled to ambient temperature and poured on broken ice containing 3 mL of cc H_2_O_2_. The filtrate was centrifuged, and the remaining solid material was washed successively with water, 30% HCl, and ethanol. Each washing step was followed by centrifugal separation. Then, the solid product was washed several times until a neutral pH was achieved. The brown ca. 1 *w*/*w*% aqueous suspensions were stored in amber bottles. For characterisation methods requiring solid probes, the freeze-dried samples were used. For the sake of simplicity, the derived graphene oxides were labelled as AF 96/97 and PM 995. Figure 1 shows the SEM images of these products.

The most relevant physicochemical properties of the obtained graphene oxides are reported in Table 1. Additional information is given in the Supplementary Materials. Most of these properties are very similar. The particle-size distribution (Figure S1) reflects the size of the pristine graphite precursors. The AF 96/97 particles are slightly thinner and their interplanar space is slightly narrower. The difference in the specific surface area values of the samples is the greatest. The detailed results of the surface chemical analysis using X-ray photoelectron spectroscopy (XPS) are summarised in Supplementary Tables S1 and S2.

### 2.2. Evaluation of the Ecotoxicity of Graphene Oxide Nanoparticles

Various measurement methods were applied, including single- and multispecies test systems at different trophic levels.

#### 2.2.1. *Aliivibrio fischeri* Bioluminescence and Enzymatic Inhibition Test Method

*Aliivibrio fischeri* is a marine, Gram-negative bioluminescent bacterium, and its emitted luminescence intensity depends on the media. When exposed to a toxic environment, the excreted light intensity decreases.

*Aliivibrio fischeri* NRRL B-111 77 strain was used for the experiments. The bacterial culture was inoculated in 40 mL of liquid Photobacterium medium 18 h before the experiments, and it was shaken at 160 rpm at 24 °C in the dark to prepare an overnight (O/N) culture. The components of the Photobacterium medium were 30 g of NaCl, 6.1 g of NaH_2_PO_4_·H_2_O, 2.75 g of K_2_HPO_4_, 0.204 g of MgSO_4_·7H_2_O, 0.5 g of (NH_4_)_2_HPO_4_, 5 g of peptone, 0.5 g of yeast extract, 3 mL of glycerol per 1 L of distilled water, and pH = 7.2 [30]. The experiments were carried out using microplate assays. The applied nanoparticles were sonicated for 15 min before usage, and a 2-fold dilution series was prepared with sterile distilled water.

Then, 50 µL of NP suspension at different concentration was transferred into the wells of the microplates, and 200 µL of O/N bacterial suspension was added to reach a final NP nominal concentration of 3.13–400 mg/L in the wells. Every treatment had 6 replicants. The control wells contained distilled water (DW) instead of the NP suspension, whilst copper sulphate was used as the positive control to detect the sensitivity of the bacterial culture. After the plates were prepared, they were incubated at 24 °C in the dark, and the luminescence intensity was measured after 30 and 120 min exposure time using a Fluostar Optima (BMG Labtech, Ortenberg, Germany) microplate reader.

To observe the toxicity of nanoparticles, a modified metabolic activity assay was performed, as described by Palomino et al. [31]. This method examines cell viability based on the mitochondrial respiratory chain of bacterial cells. The technique applies a weakly fluorescent, blue-coloured dye, resazurin (7-hydroxy-3*H*-phenoxazin-3-one-10-oxide, Alamar blue), which is reduced to highly fluorescent, pink-coloured resorufin by means of microbial respiration. The reaction takes place in viable cells’ mitochondrial respiratory chain. By measuring the change in fluorescence intensity, the number of viable bacteria can be evaluated. This experiment was carried out by adding 30 µL of 0.5 mM sterile resazurin solution into each well containing the NP/DW–bacterial suspension. Then, the plate was incubated for 15 min in the dark at room temperature, and the fluorescence intensity was measured using a Fluostar Optima (BMG Labtech) microplate reader with excitation at 544 nm and emission at 590 nm wavelength.

The effects of NPs were characterised based on the inhibition percentage (I [%]) compared to the control, which was calculated using the following equation:

I [%] = (C − S)/C × 100, (1)

where C is the value of the control and S is the value of the NP-containing sample.

#### 2.2.2. *Escherichia coli* Cell Proliferation, Enzymatic Inhibition, and ROS Detection Experiments

*Escherichia coli* ATCC 11775 Vitroids^TM^ was used to evaluate the inhibition effect of nGOs on the cell proliferation of Gram-negative bacteria. The slant cultures were maintained on LabM Luria–Bertani (LB) broth agar in the laboratory. One loopful of the colony was inoculated in 30 mL of liquid LB broth 18 h before the experiments, and it was shaken at 160 rpm at 37 °C to prepare an overnight (O/N) culture. The optical density of the O/N culture was measured at 600 nm using a SANYO SP 55 UV/VIS spectrophotometer and diluted with sterile LB broth to obtain an initial OD value of 0.095 for the tests. The bacterial suspension was 100-fold diluted by using sterile LB broth prior to the experiments. The experiments were carried out using 96-well microtiter plates, as described by the *A. fischeri* test method. A total of 50 µL of the sample was pipetted into each well from the diluted nGO suspension, and 200 µL of diluted bacterial culture was added into each well. To ensure the sensitivity of the *E. coli* suspension, antibiotics (10 µg/mL of tetracycline and streptomycin) were used as a positive control, while the control sample was sterile distilled water. The plates were incubated at 30 °C for 24 h.

To determine the cytotoxic effect of NPs, optical density (thus, cell proliferation) was measured at 630 nm using a DIALAB ELISA EL800 (Dialab GmbH, Wiener Neudorf, Austria) microplate reader. The OD was measured immediately after the preparation of the plates and at the end of the incubation time (24 h) prior to further measurements (e.g., enzymatic activity and ROS production). Enzymatic activity measurement was performed as described above.

Reactive oxygen species (ROS) measurements were carried out to determine whether the obtained GO nanoparticles had cytotoxic effect. This experiment was based on the fact that 2′,7′-dichlorodihydrofluorescein diacetate (H_2_DCFDA) is non-fluorescent until being oxidised by intracellular ROS to highly fluorescent 2′,7′-dichlorodihydrofluorescein (DCF). With this aim, 30 µL of 10 µM sterile H_2_DCFDA solution was added to each well of the microplate, and then it was stored for 30 min in the dark at 30 °C. After incubation, the fluorescence intensity of the wells was detected using a Fluostar Optima (BMG Labtech) microplate reader with excitation wavelength at 485 nm and emission at 520 nm. The ROS production was evaluated as the ratio between the fluorescence intensity values of the control and the NP-containing wells.

#### 2.2.3. SOS ChromoTest™ with Genetically Engineered *Escherichia coli*

Potentially toxic compounds can be harmful and genotoxic or may cause DNA damage, which can be investigated using a rapid detection method, the SOS ChromoTest. The SOS Chromotest was performed using the SOS ChromoTest^TM^ Kit from Environmental Bio-Detection Products Inc., as described by EBPI [32]. This method applies a novel genetically engineered *Escherichia coli* strain and measures the primary response of cells to genetic damage [33]. Two types of graphene oxides (AF 96/97 and PM 995) were tested using the SOS Chromo™ Test kit at 22.7–181.8 mg/L concentration range. Both nGO types had two subtypes, including the original and the autoclaved suspensions. A total of 10 µL of the nGO samples was pipetted into a microplate’s wells and 100 µL of engineered *E. coli* suspension was added to each sample. Then, 10% dimethyl-sulfoxide (DMSO) was used as the negative control, and the measurement was performed according to the manufacturer’s guide. The changes in colour in the wells were visually analysed, and the absorbance values of the wells were measured using DIALAB ELISA EL800 (Dialab GmbH, Wiener Neudorf, Austria) at 630 nm, and the inhibition percentage was calculated.

#### 2.2.4. Investigation of *Tetrahymena pyriformis* Proliferation and Enzymatic Activity

*Tetrahymena pyriformis* is a protozoon that lives in aquatic environments and has a pear shape. Its proliferation and enzymatic activity are great endpoints in ecotoxicological investigations. We examined whether the obtained nGO nanoparticles had toxic effect on this test organism in the 0.004–0.2 mg/L concentration range. The test was performed in 100 mL Schott bottles with 40 mL of the final volume, which contained 34.8 mL of the nutrient medium (10 g of tryptone, 1 g of yeast extract, 1 L of distilled water), 200 µL of a mixture of antibiotics (2% streptomycin, 0.2% penicillin, 1% nystatine), 4 mL of diluted nGO suspension, and 1 mL of *T. pyriformis* (10^5^ cell/mL) suspension. Every treatment had 3 replicants, and distilled water was used as the control. The reactors were incubated on a rotary shaker (160 rpm, 22 °C) for 48 h. Samples of 2 mL were collected every 24 h into sterile test tubes.

A total of 200 µL of the collected samples was pipetted into a microplate’s wells in 3 replicates, and the optical density was measured using a DIALAB ELISA EL800 microplate reader (Dialab GmbH, Wiener Neudorf, Austria) at 630 nm; then, resazurin reduction based enzymatic activity was performed as described above. Furthermore, the metabolic activity was investigated using an MTT assay (3-(4,5-dimethylthiazol-2-yl)-2,5-diphenyltetrazolium bromide). This method was performed as the resazurin measurement, but in this case, 30 µL of 1 mg/mL MTT solution was added to each well (instead of resazurin), and after 15 min incubation, the absorbance was measured at 490 nm using the DIALAB ELISA EL800 microplate reader. On the other hand, 2 µL of the sample was pipetted into a Bürker chamber (4 times) to count the number of cells using a NIKON Eclipse E400 fluorescence microscope. Then, 10 µL of formaldehyde was applied to inactivate the cells if it was necessary to be able to count them. The inhibition rate was calculated from the obtained data.

#### 2.2.5. *Panagrellus redivivus* Mortality Test

*Panagrellus redivivus* is a nematode, and its mortality can be used as an endpoint of an ecotoxicological experiment. Our examination was carried out in microplates in the 0.98–1000 mg/L nGO concentration range.

A total of 200 µL of the diluted nGO suspension, distilled water (as control), or copper sulphate (as positive control) was pipetted into the wells, and then 10 pcs of *P. redivivus* test organisms were added into each well using a pipette (2 µL of the nutrient medium). The plate was incubated at 22 °C for 72 h, and the number of the living, dead, and immobilised individuals were counted using a NIKON SMZ800 stereomicroscope after 24 and 72 h exposure time, and the inhibition rate was calculated as described above.

#### 2.2.6. *Sinapis alba* and *Triticum aestivum* Germination Rate and Root and Shoot Length Inhibition Tests

Different experiments were performed to test the effect of nGOs on plants. Mustard (*Sinapis alba*) and wheat (*Triticum aestivum*) seed germination and their root and shoot length inhibition were examined in the 0.16–100 mg/L concentration range. Graphene oxide was diluted with distilled water, and 3.5 mL of the suspension was pipetted onto cellulose filter paper in Petri dishes (Bubbles were ejected below the paper, and the suspension was dispersed). Then, 20 pcs of *S. alba* or 16 pcs of *T. aestivum* seeds were dropped onto it. Every treatment had 3 replicates, and distilled water was used as a control. The Petri dishes were closed and covered with aluminium foil, and then incubated at 22 °C for 72 h. After exposure, the root and shoot length was measured to millimetre accuracy using a ruler. The inhibition rate was calculated as described above.

#### 2.2.7. Effect of Graphene Oxide on Aquatic Microbial Community

The aim of this study was to investigate the effect of graphene oxide on a multispecies system, the microbial community of a lake. For this purpose, Biolog EcoPlate^TM^ was applied, and ROS generation was also detected. Biolog EcoPlate^TM^ is an excellent tool for microbial physiological profiling, as well as for examining the effect of nanoparticles, as described by Németh et al. [34].

The experiment was carried out using freshwater from Lake Balaton (Hungary). The samples were collected in sterile bottles at Révfülöp (46°49′36.5″ N 17°37′50.5″ E) in July 2022, and kept at 4 °C for 24 h until the start of the experiment. The experimental setup included three replicates from every treatment, and distilled water was applied as a control. Graphene oxide suspensions were diluted with distilled water and 10 mL of diluted GO suspension was added to 190 mL of the freshwater sample in a sterile 250 mL Schott bottle to reach 0.16, 0.8, 4, 20, and 100 mg/L nominal nGO concentration in each microcosm. During the experiment, the bottles were shaken at 140 rpm and kept in the dark at 22 °C. The samples were taken out on the 7th, 14th, and 28th days to monitor the effect of nGOs on microbial community. Community-level physiological profiling (CLPP) was carried out using Biolog EcoPlate^TM^. A total of 125 µL of the samples was pipetted into EcoPlate wells, and the plates were incubated at 25 °C in the dark. Absorbance of the wells was measured at 490 nm using a DIALAB ELISA EL800 microplate reader (Dialab GmbH, Wiener Neudorf, Austria) every 24 h for 168 h. Data were collected and different endpoints and diversity indices were derived as described by Németh et al. [34].

#### 2.2.8. Statistical Analysis

Data were analysed using StatSoft^®^ Statistica 13.1 (TIBCO Software, Inc., Palo Alto, CA, USA), aiming to determine whether the treatments had significant effect at the *p* < 0.05 significance level. For this purpose, Fisher’s Least Significance Difference (LSD) procedure was applied. Analysis was performed using one-way ANOVA in the case when the endpoints were measured only once (*E. coli* proliferation and enzymatic activity, *A. fischeri* enzymatic activity, and *S. alba* and *T. aestivum* root and shoot length, respectively). The homogeneity of the data was verified by means of Cochran’s C test. Repeated measures (RM) ANOVA was used when the endpoints were measured at different contact times (*A. fischeri* bioluminescence intensity, *T. pyriformis* proliferation and enzymatic activity, *P. redivivus* mortality, and the indices of the activity of freshwater microbial community, respectively). To confirm the criteria, Mauchly’s sphericity test was applied. Significant differences are marked with distinct letters on the columns in the figures.

Dose–response curves were determined using Origin^®^ 2018 (OriginLab, Northampton, MA, USA) and the effective concentrations (EC) were defined. EC_20_ is the concentration of the examined material that causes 20% inhibition of an observed endpoint.

## 3. Results

### 3.1. Toxicity of nGOs on Aliivibrio fischeri

**Figure 2:**
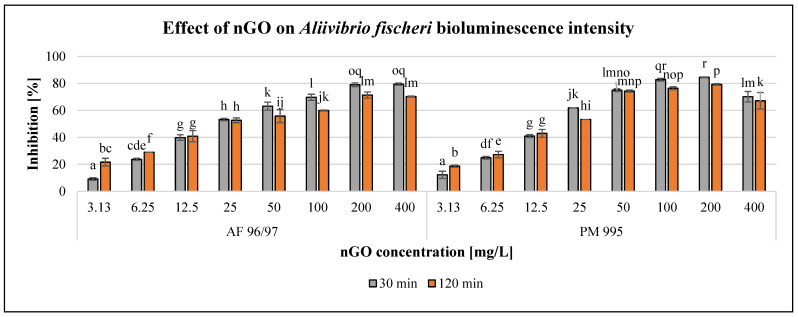
Effect of nGOs on *Aliivibrio fischeri* bioluminescence intensity. Letters on the columns indicate significant differences (level of significance: *p* < 0.05).

The observed effect of the investigated nGO nanoparticles on *Aliivibrio fischeri* bioluminescence intensity is presented in Figure 2.

The results showed a concentration-dependent negative effect of both nGO types in the investigated concentration range; however, 400 mg/L nGO PM 995 resulted in a slight decrease in the inhibition rate. Figure 2 also illustrates that incubation time has considerable influence on the effect. Within the low concentration range (3.13–6.25 mg/L), the inhibition rate was higher at 120 min exposure time than at 30 min, but even the lowest applied concentration (3.13 mg/L) of both nGO types already had a significant effect after 30 min of exposure compared to the control. At 12.5–25 mg/L nGO concentration, the differences were not significant (in the case of AF 96/97 at different exposure times), while applying higher nGO doses (>50 mg/L), the inhibition rates were smaller with longer exposure time and significant in most cases compared to the 30 min inhibition rates. The highest inhibition rates were observed after 30 min of incubation time (79.5% at 400 mg/L nGO AF 96/97, and 84.5% at 200 mg/L nGO PM 995, respectively). The EC_20_ values (Table 2) after 30 min also indicated that nGO PM 995 was more toxic than nGO AF 96/97 because the EC_20_ value was 5.01 mg/L in the case of PM 995, whilst for AF 96/97, it was 5.86 mg/L. However, after 120 min of exposure time, the EC_20_ value of nGO PM 995 was higher (4.40 mg/L), than that of AF 96/97 (4.01 mg/L); thus, it was not evident whether PM 995 would be more toxic than AF 96/97. Therefore, we could assume that nGO PM 995 had a higher toxic effect during the early stage of the experiment (30 min) because most of the *A. fischeri* cells died. Thus, at a later time (120 min), the remaining living bacterial cells could have been affected by an even higher nGO PM 995 concentration than during the early stage of the test.

**Figure 3:**
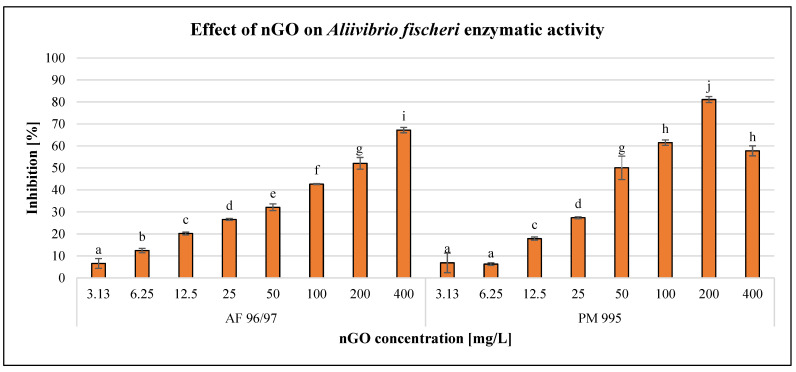
Effect of nGOs on *Aliivibrio fischeri* enzymatic activity. Letters on the columns indicate significant differences (level of significance: *p* < 0.05).

After 120 min of exposure time, the enzymatic activity of the bacterial cells was investigated using the resazurin reagent. The results are illustrated in Figure 3.

The results showed a concentration-dependent negative effect on the enzymatic activity of *A. fischeri*, although there was a decrease at 400 mg/L nGO PM 995. On the other hand, 3.13 mg/L nGO concentration caused significant inhibition for both nGO types. At lower nGO concentrations (3.13–25 mg/L), the effect was very similar for both types, but at higher doses, PM 995 had a greater inhibitory effect, except at 400 mg/L. This was confirmed by the statistical analysis, which indicated significant differences in the treatments. The highest negative effect was 81%, which was observed at 200 mg/L nGO PM 995, while in the case of AF 96/97, it was 67% at 400 mg/L.

On the other hand, the inhibition rate was lower in every case compared to the bioluminescence intensity measurements. This indicates that bioluminescence intensity detection is a more sensitive endpoint than enzymatic activity measurement. The EC_20_ values (Table 2) of AF 96/97 and PM 995 (22.86 mg/L and 16.13 mg/L, respectively) supported the above statement. Both EC_20_ values were higher than the EC_20_ values calculated based on bioluminescence intensity. These results suggest that nGO PM 995 is more toxic to *A. fischeri* than AF 995.

### 3.2. Toxicity of nGOs to Escherichia coli

The effect of GO nanoparticles on *Escherichia coli* proliferation was examined by means of OD measurement using a DIALAB ELISA EL800 microplate reader. This method did not provide reliable results since the colour of the GO NPs influenced the measured values in the wells of the microplate. This way, only the enzymatic activity investigation based on the resazurin method was applicable to this test organism since it was based on measuring fluorescence intensity.

The results of the ROS production test did not show an increase (ROS activity enhancement) but a decrease that was proportional to the GO concentration (Table S3). This suggests that the tested GO suspension does not trigger any oxidative stress reaction, but it has a cytotoxic effect through a different mechanism of action.

**Figure 4:**
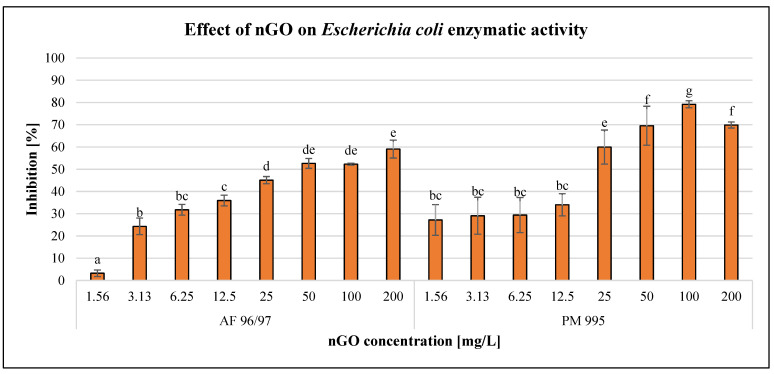
Effect of nGOs on *Escherichia coli* enzymatic activity. Letters on the columns indicate significant differences (level of significance: *p* < 0.05).

The effects of the GO nanoparticles on *Escherichia coli* enzymatic activity are illustrated in Figure 4.

The GO nanoparticles had a dose-dependent negative effect on *E. coli* enzymatic activity in the case of every treatment. PM 995 at the low concentration (1.56–12.5 mg/L) treatments showed no significant differences, since the effects were very similar. On the other hand, when applying higher concentrations (25–200 mg/L), PM 995 had a greater inhibitory effect on enzymatic activity compared to AF 96/97. The highest inhibition rate was 79% at 100 mg/L nGO PM 995, while in the case of AF 96/97, it was only 59% at 200 mg/L. The higher negative effect of PM 995 was confirmed by the statistical analysis, which showed that AF 96/97 had a significant effect at 3.13 mg/L, while PM 995 had a significant effect even at 1.56 mg/L concentration. The calculated EC_20_ values (Table 2) also indicated that PM 995 was more toxic since its EC_20_ value was 2.57 mg/L, while in the case of AF 96/97, it was 3.97 mg/L.

### 3.3. Genotoxicity of nGOs as Determined Using SOS Chromotest

The analysis of the genotoxic activity of the tested materials was carried out visually and quantitatively using photometric instrumentation. During the evaluation of genotoxicity, the turbidity and colour of the samples caused problems at 1×, 2×, and 4× sample dilutions (500–2000 mg/L concentration range). At 8× sample dilution, this interference was no longer significant, and we did not detect any genotoxic effects. According to these results of the SOS chromotest, none of the tested samples were genotoxic at 250 mg/L concentration since the induction factors (IF) were lower than 1.5.

### 3.4. Toxicity of nGOs to Tetrahymena pyriformis

**Figure 5:**
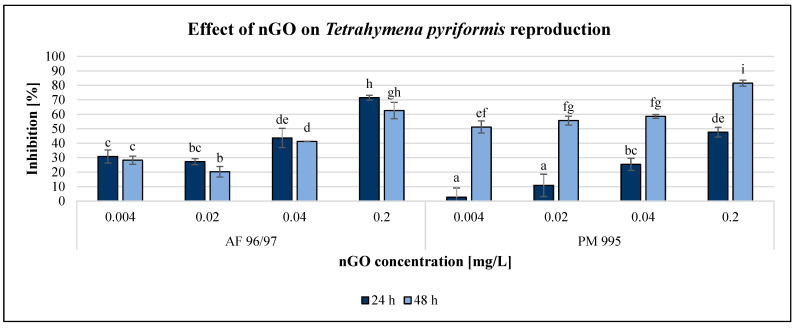
Effect of nGOs on *Tetrahymena pyriformis* reproduction. Letters on the columns indicate significant differences (level of significance: *p* < 0.05).

**Figure 6:**
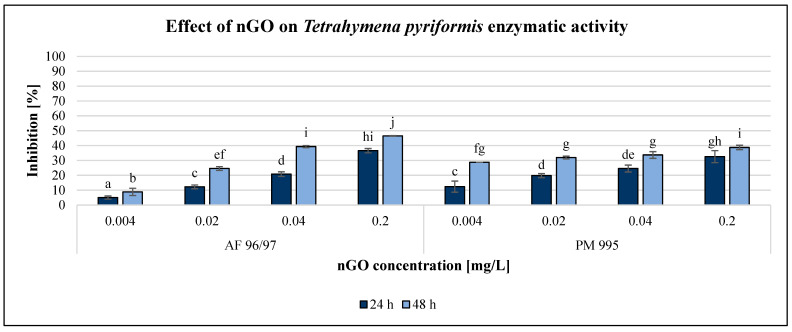
Effect of nGOs on *Tetrahymena pyriformis* enzymatic activity. Letters on the columns indicate significant differences (level of significance: *p* < 0.05).

The effect of GO nanoparticles on *Tetrahymena pyriformis* was investigated using various methods and endpoints. The OD measurement obtained using a DIALAB ELISA EL800 microplate reader was not acceptable since the colour of the GO NPs influenced the measured values. The enzymatic activity measurement based on the MTT assay was also unreliable. However, the resazurin method based on fluorescence intensity was reliably applicable for the evaluation. Furthermore, determination of the reproduction rate by counting the number of cells using a microscope was the most reliable method to evaluate the effect of GO nanoparticles. The results of the reproduction rate are illustrated in Figure 5, while the enzymatic activity is shown in Figure 6.

Both GO nanoparticles had a dose-dependent decreasing effect on the reproduction rate of *T. pyriformis*. The exposure time had a high impact on this endpoint, especially in the case of PM 995. For AF 96/97, the inhibition rate was similar after 24 and 48 h of incubation, with a slight decrease after 48 h. The inhibitory effect of PM 995 was much higher after 48 h compared to after 24 h of exposure, especially at lower concentrations. It was confirmed by the statistical analysis, which showed that after 24 h of exposure, nGO AF 96/97 had a significant effect even at 0.004 mg/L, while for PM 995, it was only at 0.02 mg/L. After 48 h, both types caused significant inhibition compared to the control even at the lowest tested nGO concentration, 0.004 mg/L. The highest observed negative effect was 81% at 0.2 mg/L nGO PM 995 after 48 h of incubation, while in the case of AF 96/97, the highest inhibition rate was 71% after 24 h at the same concentration.

The results of the enzymatic activity test showed similar pattern to the reproduction values, albeit in the case of this endpoint, the inhibition rate was always higher after 48 h than after 24 h of incubation time. Similar to the results of the *A. fischeri* test, the *T. pyriformis* enzymatic activity measurement was less sensitive than the reproduction measurement since every treatment had a lower effect on enzymatic activity than on reproduction, although the statistical analysis showed that both nGO types had a significant effect at 0.004 mg/L compared to the control even after 24 h.

However, PM 995 had significant effect on the reproduction rate only at 0.02 mg/L nGO concentration. The highest inhibition of enzymatic activity was 46% at 0.2 mg/L nGO AF 96/97 after 48 h, while in the case of PM 995, it was 39% under the same conditions. The EC_20_ values were lower in the reproduction rate investigations than in the enzymatic activity measurements at the same incubation time, except for PM 995 after 24 h of exposure (Table 2). On the other hand, the lowest EC_20_ values (0.004 mg/L) were observed for PM 995 after 48 h, both in the reproduction rate and enzymatic activity experiments.

### 3.5. Toxicity of nGOs to Panagrellus redivivus

**Figure 7:**
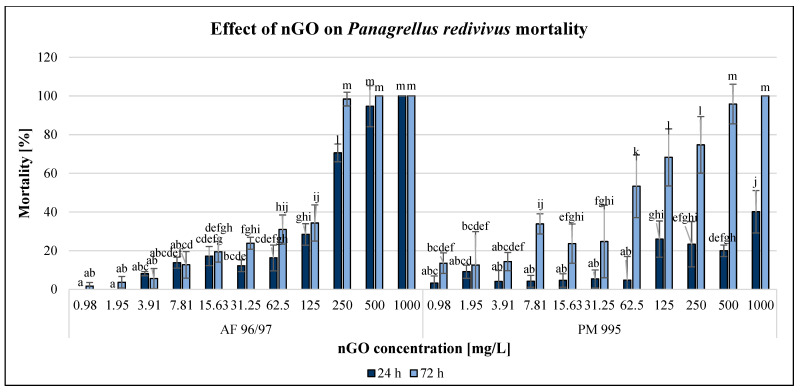
Effect of nGOs on *Panagrellus redivivus* mortality. Letters on the columns indicate significant differences (level of significance: *p* < 0.05).

The effect of GO nanoparticles was examined using *P. redivivus* mortality test. The results are illustrated in Figure 7.

Both types of GO nanoparticles had concentration- and time-dependent effects on this test organism, although there were some outliers. After applying 1000 mg/L nGOs, the mortality was 100% for both types of nGOs after 72 h; however, in the case of AF 96/97, mortality occurred even after 24 h. This type had a stronger negative effect mainly after 24 h of exposure at high concentrations (>250 mg/L), but after 72 h, the inhibition was 98% at 250 mg/L, while in the case of PM 995, it was 74% at the same concentration. On the other hand, when applying lower concentrations (<125 mg/L), the effect was higher in the case of PM 995. The lowest significant negative effects were observed in the case of AF 96/97 and PM 995 after 24 h at 7.81 and 125 mg/L nGO concentrations, while after 48 h of exposure, this was observed at 15.63 and 0.98 mg/L, respectively. Therefore, the EC_20_ values (Table 2) also indicated that after 24 h, the effect was lower in the case of AF 96/97 (79.74 mg/L), while after 72 h exposure, the EC_20_ value of PM 995 was lower (11.33 mg/L).

### 3.6. Toxicity of nGOs to Sinapis alba and Triticum aestivum

**Figure 8:**
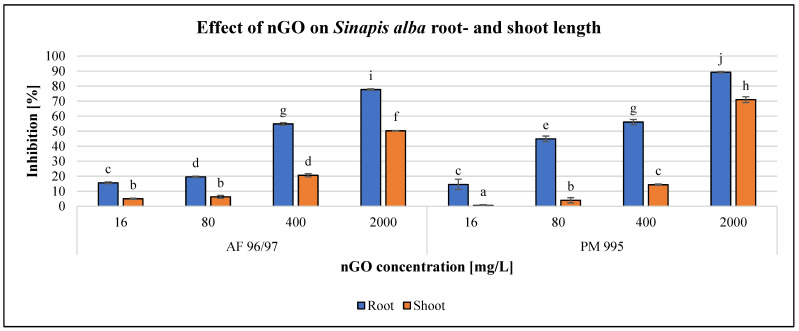
Effect of nGOs on *Sinapis alba* root- and shoot length. Letters on the columns indicate significant differences (level of significance: *p* < 0.05).

**Figure 9:**
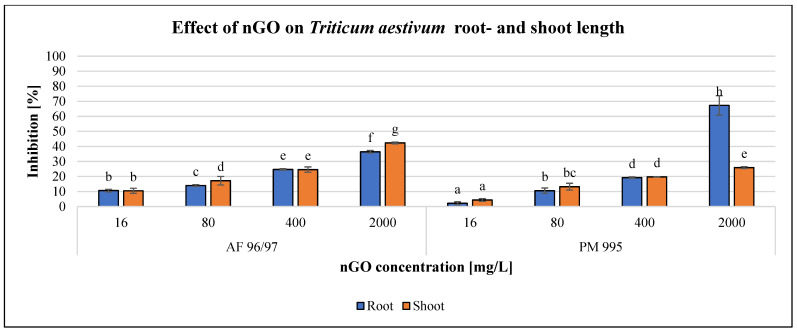
Effect of nGOs on *Triticum aestivum* root- and shoot length. Letters on the columns indicate significant differences (level of significance: *p* < 0.05).

The effects of the applied nGO particles on *S. alba* and *T. aestivum* are shown in Figure 8 and Figure 9. These nanoparticles inhibited root and shoot growth in every treatment in both plant species, and the negative effect was concentration-dependent.

For *S. alba*, the inhibition effect was higher on root growth than on shoot growth for both types of nGOs. Both types had a significant effect at 16 mg/L concentration on both measured endpoints, and the highest negative effect was observed at 2000 mg/L nGO PM 995 (89% root growth inhibition). The greatest shoot growth inhibition was 70% for the same treatment.

The highest observed inhibition values for nGO AF 96/97 were 78 and 50% in root- and shoot growth at 2000 mg/L, respectively. These results indicated that root length measurement was more sensitive than shoot length measurement. The EC_20_ values confirmed this statement, since based on the root measurement, the EC_20_ was 76.71 and 40.73 mg/L in the case of AF 96/97 and PM 995, respectively, while based on the shoot length investigation, the estimated EC_20_ of AF 96/97 and PM 995 was 380.13 and 554.01 mg/L, respectively (Table 2).

The effect of the investigated NPs on *T. aestivum* was also concentration-dependent (Figure 9), similarly to *S. alba*, but the effect was almost the same for both measured endpoints, except in the case of the highest observed inhibition of 67% at 2000 mg/L nGO PM 995, while the effect on shoot length was only 26% with the same treatment. Additionally, the inhibition rates were higher in the case of AF 96/97 compared to PM 995 at every examined nGO concentration, and the highest effect on shoot length inhibition was 42% at 2000 mg/L nGO AF 96/97. These results were in accordance with the statistical analysis, which showed that AF 96/97 had a significant effect on both measured endpoints at 16 mg/L compared to the control, while 16 mg/L PM 995 had an effect on shoot length, but the root growth was inhibited only at 80 mg/L.

The EC_20_ values of *T. aestivum* shoot- and root length (224.78 and 223.98 mg/L) (Table 2) also indicated that AF 96/97 had a similar effect on root- and shoot length. On the other hand, PM 995 had a higher effect on *T. aestivum* shoot length than root length since the EC_20_ values were 203.49 and 392.53 mg/L, respectively.

### 3.7. Toxicity of nGOs to the Microbial Community of Lake Balaton

The effect of the obtained GO nanoparticles on freshwater microbial community was investigated to determine how these NPs influenced microbial activity. Community-level physiological profiling was performed using Biolog EcoPlate^TM^, and different endpoints were calculated to describe the effect of nGOs on microbial activity.

**Figure 10:**
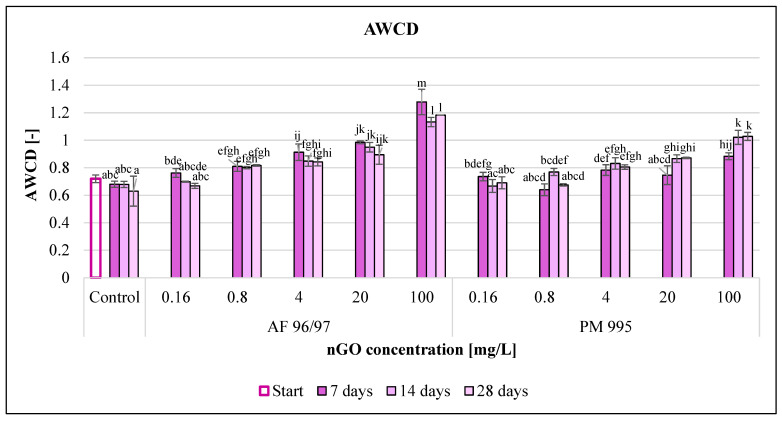
Effect of nGOs on the average well colour development value of freshwater microbial community. Letters on the columns indicate significant differences (level of significance: *p* < 0.05).

The average well colour development (AWCD) values (Figure 10) increased with incremental nGO concentrations. In the case of AF 96/97, the AWCD values increased compared to the control in every treatment, while PM 995 had a slightly negative effect at 0.16 and 0.8 mg/L after 14 and 7 days of incubation, but in the other cases, it also enhanced the AWCD values. PM 995 also had a smaller effect in every treatment compared to the other nGO type; the highest AWCD value was 1.02 at 100 mg/L after 14 and 28 days, while the AWCD value for the AF 96/97 treatment at the same concentration and after 7 days of incubation was 1.28.

The incubation time also had an impact on the AWCD values, but the results were mixed since the control and the nGO AF 96/97 treatments resulted in lower AWCD values, whilst the AWCD values of the nGO PM 995 treatments increased (except at 0.16 mg/L) with the passing of the incubation time. On the other hand, the lowest concentration showing a significant difference compared to the control was the same at every measured time points for both nGO types (0.8 and 4 mg/L for AF 96/97 and PM 995, respectively).

The measured AWCD values indicated that both nGO types significantly enhanced the microbial activity.

The results regarding substrate richness (SR) (Figure S4) had a similar pattern to the observed AWCD values. Both types of nGOs increased the number of the utilised substrates compared to the control. AF 96/97 had a higher effect at lower concentrations than PM 995. The highest SR value was 27 at 100 mg/L nGO PM 995 after 28 days of exposure time.

As the incubation time elapsed, the number of the utilised substrates was less in the case of AF 96/97 and higher with PM 995 treatments, as opposed to the AWCD values. The observed SR values also indicated an enhanced microbial activity in accordance with the AWCD values.

**Figure 11:**
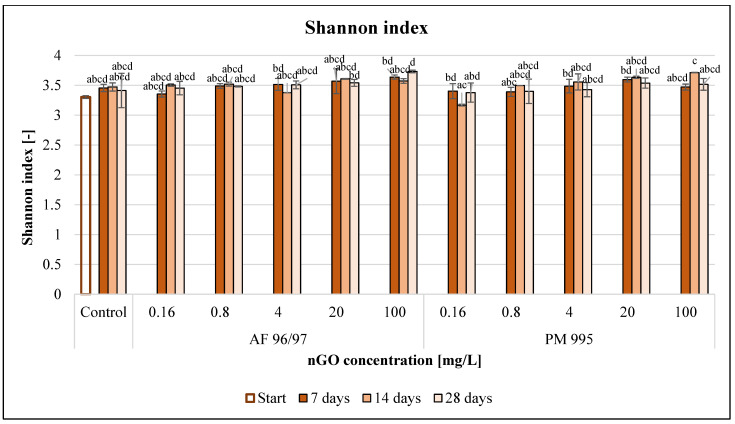
Effect of nGOs on freshwater Shannon index value. Letters on the columns indicate significant differences (level of significance: *p* < 0.05).

The Shannon index values (Figure 11) also increased for both nGO types with an increase in the applied nGO concentrations. However, a treatment of 0.16 mg/L nGO PM 995 resulted in a slight decrease at every sampling time compared to the control.

In the case of PM 995, the Shannon index values were lower than those of AF 96/97 at equal concentrations, but there were no significant differences across the treatments. Moreover, there were no observed significant effects compared to the control at any treatment concentration and time point.

There were notable differences between the McIntosh index values (Figure S5). Every treatment had an enhancing effect on this endpoint with an increase in the applied concentration. Both nGO types had a significant effect compared to the control after 7 days of incubation at 0.16 mg/L concentration, while after 28 days of incubation time, 0.16 mg/L nGO AF 96/97 treatment also had a significant influence, but PM 995 had a significant effect only at 4 mg/L. The highest McIntosh index value was 8.18 after 7 days at 100 mg/L nGO AF 96/97, and 6.42 after 28 days at the same concentration of PM 995.

**Figure 12:**
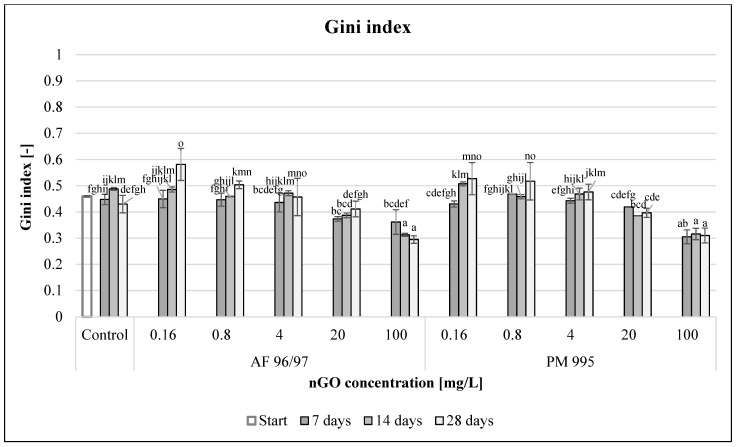
Effect of nGOs on freshwater Gini index value. Letters on the columns indicate significant differences (level of significance: *p* < 0.05).

To evaluate functional diversity, Gini index values were calculated (Figure 12). With incremental nGO concentrations, the Gini coefficient values decreased for both nGO types. In the 0.16–4 mg/L concentration range, there were significantly higher values, while at 20–100 mg/L concentrations (significantly in the latter case), the values were lower compared to the control after 28 days of incubation time. The highest Gini index values for nGO AF 96/97 and PM 995 at 0.16 mg/L concentration were 0.58 and 0.53, respectively, whilst the lowest Gini index values at 100 mg/L nGO AF 96/97 and PM 995 were 0.29 and 0.31, respectively.

These values also indicated that the obtained nGOs at high concentrations (20–100 mg/L) enhanced the functional activity of the microbial community because low Gini coefficient values indicated high functional diversity, although the high values suggested that these nGOs had a negative effect at lower (0.16–4 mg/L) concentrations.

Substrate average well colour development (SAWCD) values represent the usage rate of carbon sources (Figure S6), assuming that the total utilisation of the six substrate groups was 100%. Although there were not significant differences among the treatments, our results showed that at high nGO concentrations (100 mg/L), the metabolisation of polymers was degraded, especially in the case of PM 995, and the exposure time also had an impact because the ratio decreased with time. On the other hand, the ratio of amine usage increased slightly.

### 3.8. Determination of the Effective Concentration and PNEC Values for the Tested GO Suspensions

The ecotoxicological effect on producers and consumers was estimated using acute tests. Our results demonstrated that short-term exposure to GO suspensions could be toxic at the three trophic levels.

To determine the effective concentration (EC) values, sigmoidal curves were fitted to the inhibition percentage values calculated using the measurement data and compared to the control sample data. The nGO concentrations that caused 20% inhibition of the measured endpoint (EC_20_) were determined using the Origin^®^ 2018 software by applying logistic function fitting.

The results are shown in Table 2.

Table 2 summarises the EC_20_ values for both GO suspensions in all the applied test systems and endpoints at different contact times.

Thus, we could compare the sensitivity of the test organisms to the obtained GO NPs, and the effect of exposure time.

According to the EC_20_ values in Table 2, more toxic effects are indicated in red (and its shades), while green and its shades show lower ecotoxicity. The lower the EC_20_ value, the more toxic the nGOs.

**Table 2:** EC_20_ values [mg/L] of the applied graphene oxide suspensions in the examined test organisms. The more toxic effects are indicated in red (and its shades), while green and its shades show lower ecotoxicity. The lower the EC_20_ value, the more toxic is the nGO.

Examined Endpoint of the Test Organisms	Incubation Time	AF 96/97	PM 995
		EC_20_ [mg/L]	EC_20_ [mg/L]
*Escherichia coli* enzymatic activity	24 h	3.97	2.57
*Aliivibrio fischeri* bioluminescence intensity	30 min	5.86	5.06
	120 min	4.01	4.40
*Aliivibrio fischeri* enzymatic activity	120 min	22.86	16.13
*Tetrahymena pyriformis* reproduction	24 h	0.008	0.033
	48 h	0.011	0.004
*Tetrahymena pyriformis* enzymatic activity	24 h	0.038	0.020
	48 h	0.014	0.004
*Panagrellus redivivus* mortality	24 h	79.74	217.56
	72 h	56.09	11.33
*Sinapis alba* root length	72 h	76.71	40.73
*Sinapis alba* shoot length	72 h	380.13	554.01
*Triticum aestivum* root length	72 h	224.78	392.53
*Triticum aestivum* shoot length	72 h	223.98	203.49

The predicted no-effect concentrations (PNECs) were determined from the effective concentration values (EC_20_) and were used to calculate the risk characterisation ratios for aquatic ecosystems. This simple approach was applied to the two types of nGOs to obtain the first risk characterisation. The so-called assessment factor scheme [19] was applied to determine the PNEC_AF96/97_ and PNEC_PM995_ that were used uniformly for calculating PNECs in aquatic habitats. Based on our results obtained from the acute toxicity tests using test organisms from three trophic levels, the applied assessment factor (AF) was 1000. The PNEC_AF96/97_ = 8 ng/L and PNEC_PM995_ = 4 ng/L were determined by selecting the lowest effective concentration values (SC) and applying 1000 as an assessment safety factor (SC/AF).

The level of potential environmental risk (RCR) can be characterised by the risk characterisation ratio (RCR), which is the quotient (RCR = PEC/PNEC) of the predicted environmental concentration (PEC) and the predicted no-effect concentration (PNEC), which probably does not affect the ecosystem adversely. To determine the PEC value, the environmental concentrations reported in the literature were considered. According to the worst-case scenario approach, the highest value was used in the calculations (1.82 ng/L [20] (PEC = 1.82 ng/L)). Thus, taking this into account, the PEC/PNEC ratio for AF 96/97 nGO was 0.23 (RCR _AF96/97_ = 0.23), while for PM 995 nGO, it was 0.46 (RCR _PM995_ = 0.46). Both values are less than 1, indicating that the tested GO nanomaterials (GO AF 96/97 and GO PM 995) do not pose any risk even when assuming the worst case.

## 4. Discussion

The toxicity of two distinct forms of nGOs (AF 96/97 and PM 995) to various test organisms from diverse trophic levels (bacteria, protozoon, freshwater microbial community, plants, and invertebrate animals) in aquatic environments was examined and compared. When comparing the results of the concentration data and the effect assessment, we found correlation in most cases; higher nGO concentrations resulted in stronger negative effects. Based on the complex ecotoxicity assessment, the impact of both nGO types was characterised by calculating the effective concentration values for each test organism and the derived initial PNEC values. Finally, to characterise the potential environmental risk using a worst-case approach and conventional risk assessment, the predicted environmental concentration (PEC) was compared with the predicted no-effect concentration (PNEC).

The toxicity level of the tested GO NPs was dependent on the type and concentration of these GOs, the exposure time, and the test systems. Regarding the ecotoxicity tests, the results differed according to the test organisms, the selected endpoints, and the exposure time.

We found only small differences in terms of physicochemical properties of the tested GO nano-suspensions. The main difference was that AF 96/97 had a smaller particle size and higher specific surface area (BET: 84 m^2^/g) than PM 995 (BET: 40 m^2^/g). Thus, in some cases (e.g., in bacterial test systems), there were only minor differences in the degree of ecotoxicity between the two nGOs. However, in the case of some other applied ecotoxicological test organisms (such as *Tetrahymena pyriformis* and *Panagrellus redivivus),* the slight differences in physicochemical characteristics of the two nGOs resulted in substantial differences, both in the extent of their ecotoxicity and their temporal behaviour. Overall, it could be concluded that no clear structure–activity relationship was detected in terms of the differences in physicochemical characteristics of the tested graphene oxides and the exerted ecotoxicity, which highlighted that the test system characteristics and the interactions between the nGOs and the complex matrices significantly influenced these effects.

The outcomes of the complex ecotoxicity assessment showed that the spectrum of test organisms applied in the test batteries should encompass different trophic levels. The sensitivity of these test systems based on the EC_20_ values is as follows:

*Sinapis alba* shoot length < *Triticum aestivum* root length < *Triticum aestivum* shoot length < *Sinapis alba* root length < *Panagrellus redivivus* mortality < *Aliivibrio fischeri* enzymatic activity < *Aliivibrio fischeri* bioluminescence intensity < *Escherichia coli* enzymatic activity < *Tetrahymena pyriformis* enzymatic activity < *Tetrahymena pyriformis* reproduction

We found that ecotoxicity increased with exposure time in the most sensitive test systems, which indicated that the transformation of nGOs in these test systems could enhance their harmful effects on these representatives of the trophic chains.

The plant tests exhibited the lowest sensitivity. When comparing the effect of the two nGO types on *S. alba* and *T. aestivum*, *S. alba* proves to be more sensitive than *T. aestivum*, and root length measurement is recommended because it shows considerable sensitivity compared to shoot length measurement. Although the root EC_20_ values suggested that nGO PM 995 was more toxic than AF 96/97, the shoot EC_20_ values did not follow this finding. Both tests are suitable for the evaluation of the effect of nGOs (at high concentration levels); however, root length measurement is more suitable for use because it can detect the effect even at lower NP concentrations. Our results indicate that *S. alba* root and *T. aestivum* shoot growth measurements are moderately sensitive endpoints.

Our results are in accordance with Begum et al.’s findings [35]. These authors investigated graphene phytotoxicity to cabbage, tomato, lettuce, and spinach within the 500–2000 mg/L concentration range, and their results showed that root- and shoot growth was highly inhibited. Wang et al. [36] also found that graphene family nanomaterials (GFNs) caused a delay in seed germination and changes in plant morphology through physical, physiological, and biochemical effects (shading, mechanical injury, ROS enhancement, and modifying antioxidant enzyme activities). Zhang et al. [37] examined the effect of graphene on *Triticum aestivum* root- and shoot length within the 250–1500 mg/L concentration range, and in contrast to our results, they found improved root elongation, although there was considerable decrease in root hair production. Ren et al. [38] also reported an enhancing effect of nGOs, wherein wheat seedlings’ root growth was promoted at 100 mg/L, although inhibition was observed at higher concentrations. Liu et al. [39] described delayed germination rates of rice seeds caused by nGOs, whose effect was concentration-dependent (50 µg/mL and above), and radicle and plumule growth was inhibited. Wang et al. [40] and Zhao et al. [41] described that *Arabidopsis thaliana* seedlings accumulated nGOs in the root system but not in leaf cells after 2 weeks of exposure. Chen et al. [42] also found that GOs inhibited the germination of wheat, and at high concentrations, GOs accumulated in the root system, inducing oxidative stress. The effect of nGO-based materials on *S. alba* was studied by Roupcová et al. [43]. They found that the germination of *S. alba* seeds was inhibited when cultivated on nGO foils, but outside the foils, it was enhanced. However, the effect of nGOs was the lowest compared to other graphene-based nanomaterials. To summarise our results and the reported results in the literature, nGOs may benefit plant growth, but there are contradictory findings on this issue. For example, the effects on seed germination were explained as being due to decreased water uptake in one study [36], whilst another study reported increased seed water content [44], although both studies described enhanced ROS generation. Overall, regarding our results, we do not recommend contact between seeds and nGOs.

The *Panagrellus redivivus* mortality test also showed moderate but concentration-dependent sensitivity according to the percentage inhibition and EC_20_ values (ranging between 11.3 and 217.6 mg/L). This test organism (nematode) was representative of the animal (consumer) trophic level for ecotoxicological tests; however, the literature analysis revealed that nematode species are rarely used in ecotoxicological research on GOs.

According to the results obtained in this experiment, the performance of the two GO nanomaterials showed significant differences over time, which might be related to the behaviour of the two GO nanomaterials in the medium. The PM 995 nanomaterial resulted in higher mortality after a longer contact time (48 h) based on the EC_20_ values, while GO AF 96/97 showed a higher significant inhibitory effect after 24 h. The effect of GO nanomaterials on the organism *Panagrellus redivivus* in aquatic systems had not been studied before, so our research fills the gap from this point of view. However, the effects of zinc oxide nanoparticles (nZnO) on *Panagrellus redivivus* had been studied [45], showing that nZnO caused concentration-dependent mortality in the tested nematode species.

The ecotoxicity assessments using invertebrate animals had mainly been conducted on crustaceans (e.g., *Daphnia magna*) exposed to graphene oxide [46]. In these studies, the survival and reproduction rates were mainly investigated, while the moulting rate, enzyme activities, and gene expression were less often investigated. Fekete-Kertész et al. [47] tested more sensitive, sublethal endpoints (heartbeat rate, feeding activity, and reactive oxygen species (ROS) production) besides the conventional ecotoxicological endpoints (lethality and immobilisation). Their research demonstrated that nGOs affected *Daphnia magna* physiology, behaviour, and oxidative stress reactions.

The most sensitive endpoint was ROS production (EC_20_ = 4.78 mg/L.); thus, a key potential mechanism of the tested GOs was supposed to be via oxidative stress with reactive oxygen species, while the 20 ± 7% lethality and 10 ± 5% immobilisation effects were caused by 50 mg/L GOs. This result indicated a higher sensitivity of *Daphnia magna* to GOs than the nematode *Panagrellus redivivus.*

Regarding bacteria, most of the studies assessed the effects of NMs in terms of bacterial growth, enzymatic activity, metabolism (e.g., denitrification, nitrification, and respiration), and gene expression [46]. We investigated how the applied nGOs affected bacterial growth, enzymatic activity, and gene expression in two bacterial test systems by measuring *Aliivibrio fischeri* bioluminescence intensity and *Escherichia coli* growth and enzymatic activity. Lee et al. [48] examined the effect of nGOs on different species and found that there was no antimicrobial effect on *Vibrio* species. Domi et al. [49] studied the effect of GO nanoparticles on the luminescence of *Vibrio fischeri*. They concluded that 160 mg/L nGO concentration had no significant luminescence inhibition, but 800 mg/L nGO concentration induced 100% luminescence inhibition after 10 min of exposure. In contrast to this, our results showed that already at 3.13 mg/L, both types of nGOs had a significant effect after 30 min of exposure. The highest level of inhibition was only 84.5% at 200 mg/L concentration in the case of PM 995. Moreover, not only bioluminescence but also enzymatic activity of *Aliivibrio fischeri* was inhibited.

Liu et al. [50] found that GOs (and reduced GOs) had a high antibacterial activity against *E. coli*, with 40 μg/mL GO concentration causing 69.3% loss in *E. coli* viability, while reduced GOs resulted in 45.9% in bacterial inactivation percentage. Qiang et al. [51] found that nGOs had a strong cytotoxic effect on *E. coli*, inhibiting its growth and reproduction in a concentration-dependent manner. Our results also showed a dose-dependent negative effect on enzymatic activity, although the applied concentration range was higher and the highest inhibition was 79% at 100 mg/L nGO PM 995.

Besides the investigations using bacterial test organisms, the effect of nGOs was tested on *Tetrahymena pyriformis*, a protozoon, whose reproduction rate and enzymatic activity are great measurement endpoints in ecotoxicological experiments. Pan et al. [52] tested silver nanoparticles in the 0–10 mg/L concentration range and found that they elicited strong growth-inhibiting effects on *Tetrahymena thermophila*, mainly due to the high reactive oxygen species levels, which led to lipid peroxidation and mitochondrial dysfunction. Guo et al. [53] came to the conclusion that nanoparticles affected bacterial ingestion in *Tetrahymena thermophila* by inhibiting ATP synthesis and downregulating phagocytosis-related genes. Liao et al. [54] investigated the effects of graphene oxide on the growth, enzymatic activity, and oxidative stress of *Tetrahymena thermophila* and found that concentrations higher than 32 mg/L significantly inhibited the growth of this protozoon. Incremental GO concentrations initially increased the levels of reactive oxygen species and superoxide dismutase, but then decreased their levels, while acetylcholinesterase activity decreased. Contrary to this finding, our results showed that 0.004 mg/L nGO concentration already had a significant negative effect on the growth and enzymatic activity of *Tetrahymena pyriformis* after 24 h. Therefore, the smallest EC_20_ values were calculated for this test organism, which indicated that *T. pyriformis* was the most sensitive of the applied test organisms.

It has been demonstrated that graphene oxide-based nanoparticles adversely interfere with the normal physiology of phagocytic aquatic organisms, such as *T. pyriformis*. This high-sensitivity protozoon may be applicable for early warning and for conservative testing of the worst-case scenario.

The growing number of studies investigating the effects of GFMs on microbial communities in different environmental compartments showcases the emergence of this topic. Microbial communities from surface water bodies (rivers, lakes, estuaries, and aquariums) have been used to investigate the potential adverse effects of GFMs in small-scale batch incubation systems; however, the effects of GFMs on water bodies are still understudied [17]. In summary, the existing studies generally indicated the negative impacts of these nanomaterials on the growth of microbial communities, mostly due to oxidative stress.

Our study revealed that GO nanoparticles affected the activity and diversity of microbial community, although the effect depended on the applied concentration and nGO type. These results indicated that nGOs enhanced the microbial activity even at low concentrations, since we demonstrated that 0.16 mg/L nGO concentration could have significant effects on the examined endpoints. According to our results, nGO promoted microbial activity and functional diversity, whose effect was concentration-dependent. At low (0.16–0.8 mg/L) concentrations, we observed negative effects on the AWCD and Shannon index values in the case of PM 995. The Gini coefficient also indicated harmful effects in the case of both nGO types. On the other hand, substrate richness and McIntosh index values demonstrated a beneficial impact of these GO NPs on microbial community, even at low concentrations. Based on the SAWCD values, there were no significant differences in the utilisation rates of substrates among the treatments.

These results are in accordance with the findings of Németh et al. [34], who stated that titanium dioxide nanoparticles (nTiO_2_) did not have considerable influence on the metabolisation rate of various carbon groups, whilst zinc oxide nanoparticles (nZnO) strongly affected the utilisation rate of some substrate categories, especially polymers, amines, and phenolic compounds. Although they found that nZnO degraded the utilisation rate of these groups, our results suggested that nGOs reduced the metabolic activity of polymers but enhanced the metabolism of amines. On the other hand, nZnO had a negative effect on the microbial community, as demonstrated by other indices (AWCD, SR, Shannon, McIntosh, and Gini index values), while nGOs did not show these drawback effects; rather, they boosted the microbial activity.

Even though graphene oxide-based nanomaterials hold great promise for numerous applications, there is still a substantial knowledge gap regarding their potential environmental impacts based on environmental risk assessments [15], as environmental compartments and factors have a great influence on their fate and toxicity.

The strengths of the present study include the fact that the toxicological effects of two types of graphene oxide were evaluated using test organisms from different trophic levels. Amongst the applied ecotoxicological tests, there were single- and multispecies test systems, which provided a comprehensive evaluation of the short- and mid-term responses of the environment. On the other hand, we propose the use of simple, fast, and cheap test methods in the future to evaluate the effect of new GO (and other) nanoparticles within a low concentration range.

The novelty of our study consists of the estimation of the predicted no-effect concentration (PNEC) values for graphene oxides associated with the worst-case scenario by applying a wide range of ecotoxicological test systems, including bacteria, protozoa, nematodes, invertebrates, plants, and freshwater microbial communities. Our results are noteworthy; even when based on an environmental risk assessment with a conservative approach (the worst-case scenario approach), the tested GO suspensions do not pose any risk to the environment.

## 5. Conclusions

The results obtained in this study clearly demonstrate that a reliable characterisation of the effects of GO NMs on aquatic ecosystems requires a complex ecotoxicity test battery including organisms from different trophic levels and with various exposure routes. The toxicity level of the tested GO NMs was found to be dependent on the nGO type, route of exposure, dose, exposure time, and the applied test organism. The applied ecotoxicity toolkit is simple, inexpensive, and multitrophic and includes a wide-spectrum response to nGO substances; moreover, it delivers the results quickly (≤3 days). In addition, the whole response of the ecotoxicity toolkit was found to be satisfactory. This study demonstrated that the careful selection of the bioassays included in the design of a test battery is of utmost importance in environmental risk assessment.

The *Tetrahymena pyriformis* ecotoxicity test characterising GO-mediated effect on reproduction and enzymatic activity can be reliably used for an impact assessment of nGOs, even as an early warning system, since its effective concentration is close to the higher GO concentrations that can be measured in the environment.

Despite the increasing number of studies examining the toxicity of carbon-based nanomaterials including graphene oxide nanomaterials, the current knowledge is still insufficient, and it is extremely important to research their potentially hazardous effects indifferent ecosystems in case-by-case studies.

To carry out more relevant risk characterisation, multispecies test systems and long-term studies are needed in the future.

## Figures and Tables

**Table 1:** Properties of the tested graphene oxide samples.

Property	AF 96/97	PM 995
Concentration of the stock suspension [m/m%]	1.53	1.56
Particle-size distribution ^a^		
d10 [μm]	5.9	10.2
d50 [μm]	14.3	27.3
d90 [μm]	35.2	58.3
Specific surface area ^b,c^ [m^2^/g]	84	40
Average crystallite size ^b,d^ [nm]	8.24	6.74
Average distance of graphenic layers ^b,d^ [nm]	0.76	0.85
Average number of layers ^b,d^	11	8
*I*_D_/*I*_G_ ^b,e^	1.8	1.6
O/C of the GO derivatives [atomic %] ^b,f^	0.48	0.50
Concentration of the surface functional groups between pH values of 3 and 10 [mmol/g] ^g^	2.30	2.34

^a^ Figure S1; ^b^ measured using freeze-dried samples; ^c^ from low-temperature (−196 °C) nitrogen adsorption–desorption isotherms [26], Figure S2, Table S1; ^d^ from powder X-ray diffraction [27], Figure S3a; ^e^ from Raman spectroscopy [28], Figure S3b; ^f^ from XPS [28], Table S2; ^g^ from potentiometric titration [29].

## Data Availability

The data that support the findings of this study are available from the corresponding author upon request.

## Supplementary Materials

The following supporting information can be downloaded at https://www.mdpi.com/article/10.3390/nano13212858/s1. Table S1. Characteristics of the graphene oxide samples according to nitrogen adsorption isotherms; Table S2. Surface chemical composition of the GO samples based on X-ray photoelectron spectroscopy (XPS) and decomposition of the C1s and O1s regions (%); Table S3: Effect of nGO on *Escherichia coli* ROS production; Figure S1. Size distribution of the two graphene oxide samples. The particle-size distribution was measured from diluted aqueous suspensions using a laser-scattering particle-size distribution analyser (HORIBA LA 950 A2, Horiba, France); Figure S2. Nitrogen adsorption–desorption isotherms of the graphene oxide samples (a) and the corresponding pore-size distribution (QSDFT, slit geometry) (b) (Nova 2000e, Quantachrome, Boynton Beach, FL, USA); Figure S3. Powder X-ray diffraction (XRD) patterns (a) and Raman spectra (b) of the freeze-dried graphene oxide samples; Figure S4. Effect of nGOs on freshwater substrate richness value; Figure S5. Effect of nGOs on freshwater McIntosh index value; Figure S6. Effect of nGOs on freshwater substrate average well colour development value.
