# Supplementary material for: Ecotoxicity Assessment of Graphene Oxides Using Test Organisms from Three Hierarchical Trophic Levels to Evaluate Their Potential Environmental Risk
Source: Nanomaterials (Basel). 2023 Oct 28;13(21):2858. doi: 10.3390/nano13212858 (PMC10649827; doi:10.3390/nano13212858)
Supplement: Supplementary file 1 [file nanomaterials-13-02858-s001.zip › nanomaterials-2618770-supplementary.docx]

**SUPPLEMENTARY MATERIALS**

NANOMATERIALS

Ecotoxicity Assessment of Graphene Oxides Using Test Organisms from Three Hierarchical Trophic Levels to Evaluate Their Potential Environmental Risk

Imre Németh ^1^, Krisztina László ^2^, Anna Bulátkó ^2^, Emese Vaszita ^1^ and Mónika Molnár ^1,^*

1. Department of Applied Biotechnology and Food Science, Faculty of Chemical Technology and Biotechnology, Budapest University of Technology and Economics, H-1111 Budapest, Hungary
2. Department of Physical Chemistry and Materials Science, Faculty of Chemical Technology and Biotechnology, Budapest University of Technology and Economics, H-1111 Budapest, Hungary

***** Correspondence: molnar.monika@vbk.bme.hu

**Table S1. Characteristics of the graphene oxide samples from nitrogen adsorption isotherms**

| **Sample** | **Surface area [m^2^/g]** | **Total pore volume [cm^3^/g]** |
| --- | --- | --- |
| **AF 96/97** | 84 | 0.071 |
| **PM 995** | 40 | 0.051 |

**Table S2. Surface chemical composition of the GO samples from X-ray photoelectron spectroscopy (XPS) and decomposition of the C1s and O1s regions (%)**

| **Sample** | **C** | **O** | **S** | **C1** | **C2** | **C3** | **C4** | **C5** | **O1** | **O2** | **O3** |
| --- | --- | --- | --- | --- | --- | --- | --- | --- | --- | --- | --- |
|  | atomic % | | | | | | | | | | |
| **AF 96/97** | 66.5 | 32.0 | 1.5 | 23.0 | 5.6 | 30.2 | 5.3 | 2.3 | 11.1 | 17.8 | 3.1 |
| **PM 995** | 65.6 | 33.1 | 1.3 | 19.8 | 5.5 | 33.2 | 5.1 | 2.0 | 10.2 | 19.2 | 3.7 |

Assignation of the chemical states:

Component Binding energy (eV) Chemical states

C1 284.3 ± 0.1 sp2 C=C aromatic

C2 285.1 ± 0.2 sp3 C-C amorphous, aliphatic

C3 286.4 ± 0.2 C‒O‒C ether, epoxy, C‒OH hydroxyl

C4 287.7 ± 0.2 C in C=O carbonyl

C5 288.7 ± 0.2 C in O=C‒O group

O1 531.5 ± 0.2 O in carbonyl group

O2 532.6 ± 0.2 O in ether and epoxy, OH in alcohol, C=O in ester

O3 533.5 ± 0.2 C-O-C in ester, OH in carboxyl

**Table S3.** Effect of nGO on *Escherichia coli* ROS production

| Sample | Concentration [mg/L] | ROS inhibition [%] |
| --- | --- | --- |
| **AF 96/97** | 1.56 | 8.48 (± 1.28) |
|  | 3.13 | 12.66 (± 0.96) |
|  | 6.25 | 17.77 (± 0.74) |
|  | 12.5 | 22.77 (± 0.61) |
|  | 25 | 30.53 (± 0.29) |
|  | 50 | 38.70 (± 0.83) |
|  | 100 | 47.75 (± 1.22) |
|  | 200 | 62.88 (± 2.37) |
|  |  |  |
| **PM 995** | 1.56 | 7.44 (± 0.91) |
|  | 3.13 | 8.24 (± 0.38) |
|  | 6.25 | 11.88 (± 0.77) |
|  | 12.5 | 17.80 (± 0.83) |
|  | 25 | 27.19 (± 1.05) |
|  | 50 | 38.75 (± 1.11) |
|  | 100 | 52.79 (± 1.66) |
|  | 200 | 69.67 (± 1.08) |

**Figure S1.** Size distribution of the two graphene oxide samples. The particle size distribution was measured from diluted aqueous suspensions using a laser scattering particle size distribution analyzer (HORIBA LA 950 A2, Horiba, France)

|  |  |
| --- | --- |
| **a** | **b** |

**Figure S2.** Nitrogen adsorption-desorption isotherms of the graphene oxide samples **(a)** and the corresponding pore size distribution (QSDFT, slit geometry) **(b)** (Nova 2000e, Quantachrome, Boynton Beach, FL, USA)

|  |  |
| --- | --- |
| **a** | **b** |

**Figure S3.** Powder X-ray diffraction (XRD) patterns **(a)** and Raman spectra **(b)** of the freeze dried graphene oxide samples

**Figure S4.** Effect of nGO on freshwater substrate richness value. Letters on the columns indicate significant differences (level of significance: p < 0.05).

**Figure S5.** Effect of nGO on freshwater McIntosh index value. Letters on the columns indicate significant differences (level of significance: p < 0.05).

**Figure S6.** Effect of nGO on freshwater substrate average well colour development value.
